# Dysfunctional miRNA-Mediated Regulation in Chromophobe Renal Cell Carcinoma

**DOI:** 10.1371/journal.pone.0156324

**Published:** 2016-06-03

**Authors:** Xiaohan Sun, Junying Zhang

**Affiliations:** 1 School of Computer Science and Technology, Xidian University, Xi'an, Shaanxi, P. R. China; 2 College of Mathematics and Information Science, Weinan Normal University, Weinan, Shaanxi, P. R. China; National Institutes of Health, UNITED STATES

## Abstract

Past research on pathogenesis of a complex disease suggests that differentially expressed message RNAs (mRNAs) can be noted as biomarkers of a disease. However, significant miRNA-mediated regulation change might also be more deep underlying cause of a disease. In this study, a miRNA-mediated regulation module is defined based on GO terms (Gene Ontology terms) from which dysfunctional modules are identified as the suspected cause of a disease. A miRNA-mediated regulation module contains mRNAs annotated to a GO term and MicroRNAs (miRNAs) which regulate the mRNAs. Based on the miRNA-mediated regulation coefficients estimated from the expression profiles of the mRNA and the miRNAs, a SW (single regulation-weight) value is then designed to evaluate the miRNA-mediated regulation change of an mRNA, and the modules with significantly differential SW values are thus identified as dysfunctional modules. The approach is applied to Chromophobe renal cell carcinoma and it identifies 70 dysfunctional miRNA-mediated regulation modules from initial 4381 modules. The identified dysfunctional modules are detected to be comprehensive reflection of chromophobe renal cell carcinoma. The proposed approach suggests that accumulated alteration in miRNA-mediated regulation might cause functional alterations, which further cause a disease. Moreover, this approach can also be used to identify diffentially miRNA-mediated regulated mRNAs showing more comprehensive underlying association with a disease than differentially expressed mRNAs.

## Introduction

MiRNAs are important gene regulators associated with a wide variety of functions. Their dysregulation has been discovered to be the genetic cause of many complex diseases and cancers [1]. Many pathogenic studies focus on the alteration in miRNA regulation and its influence on mRNA expression. For example, Nandini Nair *et al*. identified the distinct patterns of some circulating miRNAs expression [2], Yun Xiao *et al*. identified dysfunctional miRNA-mRNA regulatory modules [3], and Tao Huang et al. identified dysfunctional gene sets in lung cancer [4]. In these studies, miRNA regulation is viewed as the cause of changed mRNA expression, but not the cause of dysfunction [5]. However, in our opinion, the remarkable alteration in miRNA regulation associated with some specific functions might be the underlying cause of a disease even with unchanged mRNA expression.

Renal cell carcinoma (RCC) is the most common neoplasm of the adult kidney and chromophobe renal cell carcinoma (ChRCC) is a rare subtype of RCC. Some studies show miRNAs simultaneously regulate both oncogenes and tumor supressors in ChRCC [6], which results in the inconsistency between mRNA and miRNA expression and miRNA-mediated regulation. Moreover, the long-term outcomes of ChRCC are much more variable than those of other RCC subtypes [7], and miRNAs can be used as bio-signatures of different RCC subtypes [8]. Therefore, investigation of tumor-specific miRNA-mediated regulation is important to understand the pathogenesis of ChRCC.

In this study, we propose an approach to identify dysfunctional miRNA-mediated regulation modules (MMRMs) by comparing miRNA-mRNA regulation between tumor and normal samples. A function in our study corresponds to a Gene Ontology term (GO, a bioinformatics resource about gene-product function http://www.geneontology.org) [4, 9]. A miRNA-mediated regulation module is a GO-term related module which contains mRNAs annotated to a GO term and miRNAs which regulate the mRNAs. Based on expression profiles of miRNAs and mRNAs, the miRNA-mediated regulation coefficients are estimated by linear regression, and a SW (single regulation-weight) value is designed to estimate cumulative difference of the regulation coefficients of an mRNA. A dysfunctional MMRM is then detected by the SW values in the module showing statistically significant difference between tumor and normal samples. We apply the identification approach on ChRCC as an example. It identifies 70 dysfunctional miRNA-mediated modules from 4381 modules. The designed SW value can also be applied to identify the underlying causal mRNAs which undergo significant miRNA-mediated regulation alteration. The identified dysfunctional MMRMs and differentially miRNA-mediated regulation mRNAs show relevance to comprehensive symptoms of ChRCC.

## Materials and Methods

**Fig 1:**
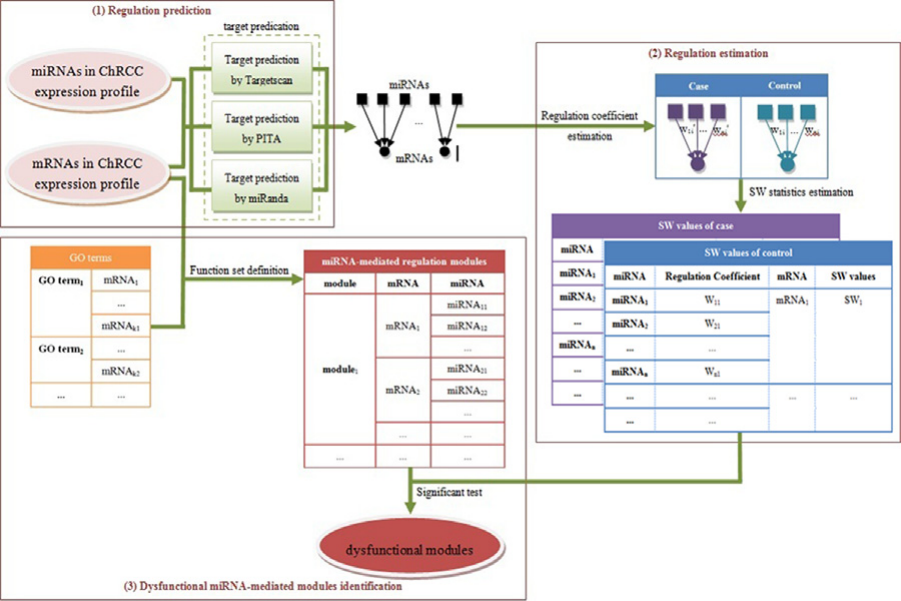
Flowchart of the approach. (1) Regulation relationship between a miRNA and an mRNA is predicated. (2) Regulation coefficients are estimated, and SW values are computed. (3) MMRMs are created and dysfunctional ones are identified.

Our approach is used to identify dysfunctional MMRMs, in which, miRNA-mediated regulation show statistically significant difference between tumours and normal samples. There are three stages for identifying dysfunctional MMRMs (Fig 1): (1) predicting regulation relationship between miRNAs and mRNAs associated with a disease (ChRCC in the study), (2) estimating the regulation coefficient of an mRNA and an miRNA and illustrating miRNA regulation alteration of an mRNA by a designed SW value, and (3) creating MMRMs according to GO terms and identifying the dysfunctional ones. We apply this approach on expression profiles of miRNAs and mRNAs of ChRCC, and identify 70 dysfunctional MMRMs.

### Prediction of miRNA-mRNA regulation

The expression profiles of miRNAs and mRNAs associated with ChRCC are downloaded from The Cancer Genome Atlas (TCGA, the NIH research program, http://cancergenome.nih.gov/). There are 25 control and 66 case samples from TCGA cohort which covers 1046 miRNAs and 20531 mRNAs (S1 and S2 Files).

The miRNA-mRNA regulation relationship can be predicted by available prediction software. There have been various resources of target mRNA predictions, which produce different results [10, 11]. A common solution to decrease the false positives is to cross check multiple algorithms to get an additional layer of confidence [10]. We choose Targetscan (http://www.targetscan.org/cgi-bin/targetscan), PITA (http://genie.weizmann.ac.il/index.html), and miRanda (http://www.microrna.org/microrna/home.do) to predict targets due to their good performance in detecting the previously validated targets [11]. Only the target mRNAs predicted by at least two algorithms are believed reliable. This way, we obtain 149181 miRNA-mRNA relationships between 289 associated miRNAs and 11458 associated mRNAs.

### Estimation of regulation coefficients

**Fig 2:**
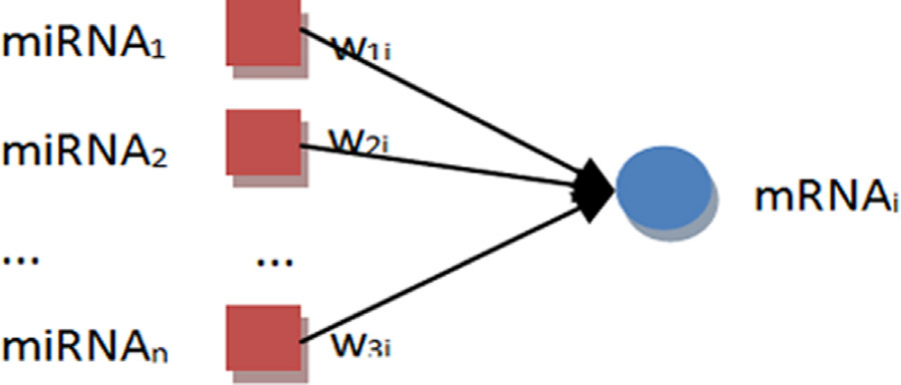
Regulation coefficients estimation between multiple miRNAs and a target mRNA. The dependent variable is an m-by-1 vector (m is the number of samples), in which each value is the expression of an mRNA sample. The explanatory variables is a m-by-n matrix, in which each column is the expression values of a miRNA across all the samples, and n is the number of miRNAs which regulate the mRNA. A n-by-1 vector [*w*_11_ *w*_21_ ⋯ *w*_*n*1_]^*T*^ is then returned, and each value in the vector is a regulation coefficient.

The miRNA-mRNA regulation strength can be measured by a regulation coefficient estimated by linear regression based on the expression profiles of miRNAs and mRNAs. Linear regression is an approach for modelling the relationship between a scalar dependent variable and one or more explanatory variables. The column vector of an mRNA expression is taken as the dependent variable, and the matrix of expression of the miRNAs is taken as explanatory variables. Multiple linear regressions here are used to estimate the regulation coefficients between an mRNA and multiple miRNAs (Fig 2).

Because an mRNA is regulated by multiple miRNAs, and the regulation change of an mRNA cannot be completely illustrated until investigating all the regulation changes of miRNAs which regulate the mRNA, we design an SW value to measure the regulation change of an mRNA. The value is defined as SW=∑i=1n(wi−wi')2n where [*w*_*i*_] and $w_{i}^{\prime }$ are the regulation coefficient vector of respective case and control samples, and n is the number of miRNAs which regulate an mRNA. We divide ∑i=1n(wi−wi')2 by n to balance the contribution from the large and small regulation coefficients.

### Identification of dysfunctional MMRMs

GO term is a name indicating the domain to which the term belongs and describing the function of gene products. We download mRNA association files of Homo sapiens from the Gene Ontology (http://geneontology.org/page/download-annotations), and obtain 13812 distinct GO terms and the mRNAs annotated to these terms (S3 File). Corresponding to a GO term, an MMRM associated with ChRCC is created and it includes the associated mRNAs annotated to the GO term and the associated miRNAs predicted to regulate the mRNAs [4]. Finally, we get 11919 associated MMRMs.

The dysfunctional MMRMs are the modules which contain mRNAs showing statistically significant difference in SW values. Due to the graph structure of GO, GO terms in different hierarchy contain different numbers of gene products, and MMRMs thus contain different number of mRNAs. With the SW value of each associated mRNA at hand, we perform t-test to determine if the SW values in a module are significantly different between case and control. t-test is relatively robust to moderate violations of the normality assumption for moderately large samples [12], hence it is adopted here because the number of mRNAs in a module is not very large. By t-test with significance level of 0.01, we identify 70 modules as dysfunctional MMRMs associated with ChRCC.

## Results

The 70 identified dysfunctional MMRMs correspond to 70 GO terms which cover 30 cellular components, 14 molecular functions and 26 biological processes (Table 1), most of them are closely associated with anatomical structure morphogenesis, cellular developmental process, blood pressure *etc*.

**Table 1:** Identified Dysfunctional MMRMs.

**Cellular component**	
**components of extracellular region part**	GO:0005577, GO:0005578, GO:0005579, GO:0005581, GO:0005582, GO:0005583, GO:0005584, GO:0005585, GO:0005586 GO:0005588, GO:0005589, GO:0005590, GO:0005591, GO:0005592, GO:0005594 GO:0005595, GO:0005596, GO:0005597, GO:0005600, GO:0005614, GO:0005615
**components of cell**	GO:0005622, GO:0005623
**components of multiple cell**	GO:0005576, GO:0005587, GO:0005604, GO:0005605, GO:0005608, GO:0005610
**Molecular function**	
**protein binding**	GO:0005515, GO:0005516, GO:0005518, GO:0005519, GO:0005520, GO:0005521, GO:0005522, GO:0005523
**Sugar binding**	GO:0005534, GO:0005536, GO:0005537, GO:0005524, GO:0005528, GO:0005539
**Biological process**	
**Cell fate**	GO:0001708, GO:0001709, GO:0001710
**blood pressure**	GO:0001711, GO:0001714, GO:0001976, GO:0001984, GO:0001987, GO:0001991, GO:0001994, GO:0002001, GO:0002002, GO:0002003, GO:0002005, GO:0002017, GO:0002018, GO:0003085
**heart contraction**	GO:0001985, GO:0001986, GO:0001996, GO:0001997
**epithelium**	GO:0002009, GO:0002011
**lymphangiogenesis**	GO:0001946
**response to amphetamine**	GO:0001975
**mesoderm formation**	GO:0001707

## Discussion

### Dysfunctional MMRMs

An MMRM includes multiple mRNAs and an mRNA is regulated by multiple miRNAs, therefore, the identified dysfunctional MMRMs are the comprehensive reflection of ChRCC. Many of the identified MMRMs do not directly involve renal cells or relevant products, but they are associated with functions of kidney, response to drugs and Birt-Hogg-Dubé (BHD) syndrome. Kidneys participate in regulating acid-base balance, extracellular fluid volume, blood pressure *etc*., which are partially overlapped with the identified dysfunctional MMRMs. Second, amphetamine diet pills increase the risk for RCC [13]. Third, BHD is a human autosomal dominant genetic disorder that can cause susceptibility to kidney cancer, and people over 20 years of age with BHD syndrome have an increased risk of developing slow-growing ChRCC[14, 15].

### Differentially miRNA-mediated regulated mRNAs

An mRNA expression could remain unchanged even if the mRNA undergoes dramatic alteration in miRNA regulation because some miRNAs prompt the mRNA expression but others could repress it. Therefore, the Differentially miRNA-mediated regulated mRNAs should be relevant to ChRCC in a comprehensive and indirect way. SW value is a measure of miRNA-mediated regulation difference, and it can be used to identify the differentially miRNA-mediated regulated mRNAs. To estimate statistical significance of a SW value, we perform 1000 permutation tests on all the samples of each mRNA, and compute a SW value for each permutation. Based on the 1000 SW values, the p-value of an SW value can be obtained. We thus get 148 mRNAs whose p-value< = 0.01 (S4 File). Among the 148 identified mRNAs, *BPGM* is unregulated in ChRCC [16], some mRNAs have been reported to be associated with renal diseases. For example, *MDM2*, *SLC16A3*, *LFT etc*. show expression change in renal cancer [17–20]; *SLC22A7*, *ACHE*, *BMP4 etc*. are associated with renal function [21–24]. Moreover, some identified mRNAs are associated with cell growth and differentiation. For example, *GAB3*, *PTPN7*, *PTPN11*, *FEG6*, *HRK etc*. regulate cell differentiation, growth, survival, apoptosis, proliferation *etc*. [25–29]. Most differentially miRNA-mediated regulated mRNAs are relevant to comprehensive symptoms of ChRCC and they might be underlying cause of ChRCC.

Significance Analysis of Microarrays (SAM) is a method for identifying differentially expressed mRNAs from mRNA expression profile. Here we adopt it finding 258 differentially expressed mRNAs. We compare the 148 mRNAs identified based on SW value with the 258 mRNAs identified by SAM and find two overlap mRNAs (*C7* and *TPT1*). The few overlap genes explain the assumption that the mRNAs undergoing major miRNA-regulation change might present stable expression.

GO enrichment analysis tool (http://geneontology.org/page/go-enrichment-analysis) is applied on the 158 identified mRNAs based on SW value to find the functions that the mRNAs enrich. Separately taking the complete biological process, molecular function, cellular component annotation data sets as background, we find 138 genes overrepresented in biological process, 3 genes in growth hormone, 133 genes in receptor binding, 132 genes in cell and 145 genes in cell part and cellular component. The overrepresented terms are higher in the hierarchical structure of GO terms, which deduces that miRNA-mediated dysregulation could have extensive effect on mRNAs.

## Conclusion

Differentially expressed mRNAs are naturally viewed as biomarkers of a complex disease, but other alterations also contribute to a disease, including miRNA-mediated regulation alteration. In this approach, we create MMRMs, and from which we identify the dysfunctional ones. We apply the approach on mRNA and miRNA expression profiles of ChRCC and identify 70 dysfunctional MMRMs. In addition, SW values can also be used to identify differentially miRNA-mediated regulated mRNAs. The proposed approach provides a novel view of mechanics of disease: miRNA regulation involves in a disease not only by affecting mRNA expression but also by miRNA dysregulation itself.

## Supporting Information

S1 File miRNA expression profile downloaded from TCGA. (ZIP)

S2 File mRNA expression profile downloaded from TCGA. (ZIP)

S3 File Genes in GO. The genes in this file are those annotated to GO terms.
(XLS)

S4 File Significant genes based on SW. The genes in this file are the differentially miRNA-mediated regulated genes identified based on SW values.
(XLS)
